# Managing Nasal Ischemia and Necrosis Post‐Triamcinolone Injection: A New Case Series

**DOI:** 10.1155/crot/2223358

**Published:** 2026-07-03

**Authors:** Shahriar Nazari, Nima Hadadian, Foroohe Bayatparidary, Seied Omid Keyhan, Mohammadamin Damsaz, Nabil Fakih-Gomez, Mohammad Reza Pourani

**Affiliations:** ^1^ Department of ENT and Head and Neck Surgery, BMI Hospital, Tehran, Iran; ^2^ Private Practice, Zibaban Clinic, Management and Research of Complications Clinic, Tehran, Iran; ^3^ School of Medicine, Ahvaz Jondishapur University of Medical Sciences, Ahvaz, Iran; ^4^ Department of Oral & Maxillofacial Surgery, College of Medicine, University of Florida, Jacksonville, Florida, USA, ufl.edu; ^5^ Maxillogram Maxillofacial Surgery, Implantology and Biomaterial Research Foundation, Istanbul, Turkey; ^6^ Department of Facial Plastic & Cranio-Maxillo-Facial Surgery, Fakih Hospital, Khaizaran, Lebanon; ^7^ Department of Surgery, University of Salamanca, Salamanca, Spain, usal.es

**Keywords:** nasal ischemia, necrosis, rhinoplasty, triamcinolone

## Abstract

Nasal ischemia following cosmetic injections has been reported in several cases. Postsurgery triamcinolone administration in the nasal region is a common practice to reduce inflammation and address pollybeak deformity. While cases of retinal artery occlusion following triamcinolone injection have been documented, this case series presents the first reported instance of nasal necrosis resulting from triamcinolone injection. This article explores the management strategies and therapeutic approaches for ischemia following triamcinolone administration. Four cases of nasal ischemia following triamcinolone injections administered postrhinoplasty are documented. The first case presented a young female who experienced ischemia with involvement of the columellar artery, leading to reduced blood flow in the dorsal and lateral nasal arteries. The remaining cases were patients who sought reconstructive surgery due to nasal necrosis caused by triamcinolone injections. Triamcinolone injection in the nasal region can lead to ischemia. Treatment options should be customized to each patient’s condition. The THIS and FAT protocol with toxins (botulinum neurotoxin), high‐dose hyaluronidase, and injectable platelet‐rich fibrin (iPRF), solid platelet‐rich fibrin (sPRF) dressings, and antibiotics as well as hyperbaric oxygen therapy (HBOT) can enhance tissue recovery, minimize scarring, and improve the overall outcome.

**Key Clinical Message**
 Triamcinolone injection in the nasal region can lead to ischemia. Treatment options should be customized to each patient’s condition. High‐dose hyaluronidase (HYAL), botulinum neurotoxin, and injectable platelet‐rich fibrin (iPRF) injections promote neovascularization. In addition, solid platelet‐rich fibrin (sPRF) dressings and hyperbaric oxygen therapy (HBOT) can enhance tissue recovery, minimize scarring, and improve the overall outcome.

## 1. Introduction

Nasal ischemia following esthetic injections has been a recognized complication since the late 19th century. Ischemia induced by hyaluronic acid (HA) fillers is well‐documented, with established treatment and management protocols [1]. However, the potential for triamcinolone acetonide injections to cause nasal ischemia remains unclear. Triamcinolone acetonide is commonly used to reduce swelling and prevent skin thickening following rhinoplasty [2].

Although rare, cases of ischemia following triamcinolone administration have been reported, particularly in the treatment of carpal tunnel syndrome (CTS). The proposed mechanisms for ischemia include intra‐arterial injection, arterial wall dissection, or reactive vasospasm triggered by steroid suspension particles [3].

This article presents a case series of postrhinoplasty vascular occlusion resulting from triamcinolone administration, leading to nasal ischemia and subsequent necrosis. We describe the clinical presentation, diagnostic challenges, and therapeutic strategies employed in the THIS and FAT protocol (neurotoxin, high‐dose HYAL, iPRF, and medications) to manage these adverse events [4]. We aim to introduce a comprehensive therapeutic approach for patients experiencing similar complications following triamcinolone injection.

### 1.1. Case Examination

A patient with acute ischemia was referred to the clinic for treatment (Case 1). Additionally, three patients who developed necrosis with subsequent scarring sought reconstructive surgery through online or in‐person consultations (Cases 2, 3, and 4). The study adheres to all relevant legal and regulatory requirements governing the use of patient data in scientific studies. All data collected for this study remains confidential. The data are securely stored, accessible only to authorized personnel, and used exclusively within the established ethical guidelines. Additionally, informed consent was obtained from all participants to share their photos.

### 1.2. Case Presentation

#### 1.2.1. Case 1

**FIGURE 1:**
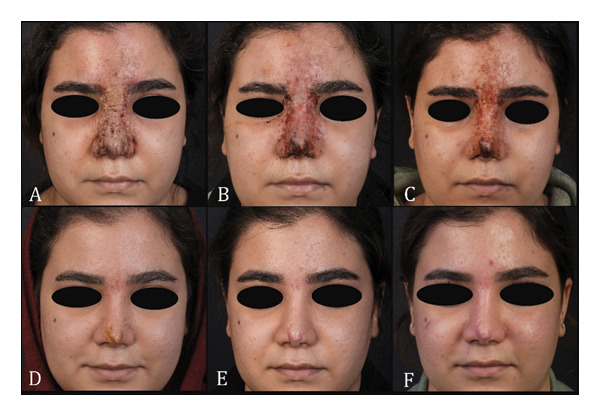
(A) Clinical presentation 54 h post‐triamcinolone injection, showing livedo reticularis and bacterial bioburden spreading toward the forehead. (B) Condition after 2 days of treatment with hyaluronidase, dysport, injectable platelet‐rich fibrin (iPRF), debridement, solid platelet‐rich fibrin (sPRF), and hyperbaric oxygen therapy. (C) Marked reduction in edema, erythema, and inflammation 4 days after treatment. (D) Significant improvement of the lesion at 4 weeks. (E) Follow‐up visit at 4 months. (F) Near‐complete resolution with fair scar at 7 months.

A 19‐year‐old female, who had undergone rhinoplasty three months earlier, presented for a routine monthly follow‐up. During the visit, she received an injection of 10 mg triamcinolone (TriamHEXAL 40 mg, HEXAL AG), diluted in 1 cc of normal saline, at the nasal tip. Immediately postinjection, pallor of the nose was observed, prompting a vigorous 30‐min massage to alleviate the vascular compromise. Despite this intervention, her condition worsened over the next 2 days, with the development of livedo reticularis and bacterial bioburden spreading toward the forehead. She was referred to the clinic 54 h after the triamcinolone injection (Figure 1A).

Upon arrival, the patient exhibited severe pain, swelling, and pus‐filled sores with areas of skin discoloration. The ischemic area measured approximately 50 cm^2^, with a four‐second capillary refill time (CRT). Livedo reticularis and bacterial bioburden were present on the nose, glabella, and central forehead. Visual acuity, visual field, and olfactory nerve examinations were normal.

Doppler ultrasound revealed reduced blood flow in the columellar, lateral nasal, and dorsal nasal arteries bilaterally, with complete occlusion of the columellar artery. Immediate treatment included 50 IU of Dysport (Abobotulinumtoxin A) injecting throughout the entire ischemic area. Then, 1500 IU of HYAL (Hyalase Wockhardt UK Ltd) was injected 2 times with an interval of 20 min. Afterward, for the wound management, the iPRF was injected. The patient was prescribed clindamycin 300 mg three times daily, levofloxacin 750 mg once daily for broad‐spectrum coverage, and aspirin 80 mg daily for 7 days. The following day, debridement, iPRF, SPRF, and HBOT were performed (Figure 1B).

The patient completed 15 HBOT sessions at 2.3 bar pressure (SECHRIST H‐CHAMBER), lasting approximately 105 min without breaks. Daily evaluations showed reduced edema, erythema, and inflammation by Day four (Figure 1C). Livedo reticularis resolved by Day six. By Day 12, most nasal skin had improved significantly, except for the tip and glabella, indicating partial restoration of blood flow.

After 4 weeks, two atrophic scars remained on the glabella and nasal tip. CRT was normal, and erythema had fully subsided in previously affected areas (Figure 1D). Subsequent follow‐up visits demonstrated significant improvement (Figure 1E,F).

#### 1.2.2. Case 2

**FIGURE 2:**
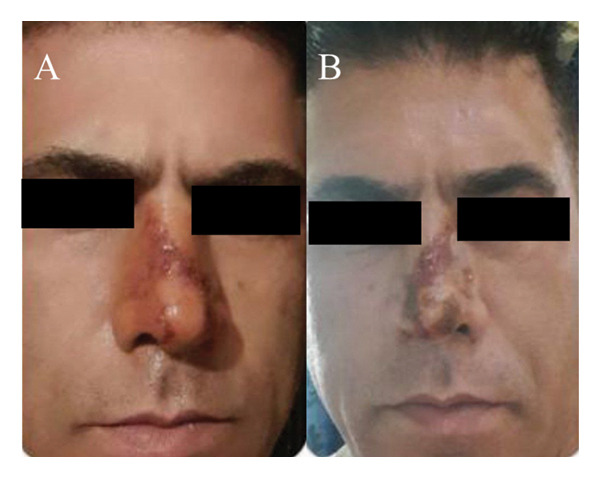
(A) Clinical presentation of livedo reticularis and ischemia following triamcinolone injections. (B) Eschar formation and full‐thickness necrosis occurred a week after the triamcinolone injection, and the patient sought consultation.

A 42‐year‐old male patient received triamcinolone injections three months after rhinoplasty to manage postoperative edema. Shortly after the injection, he developed a livedo reticularis pattern, followed by tissue necrosis. Seeking reconstructive options, the patient consulted online for surgical evaluation and management (Figure 2).

#### 1.2.3. Case 3

**FIGURE 3:**
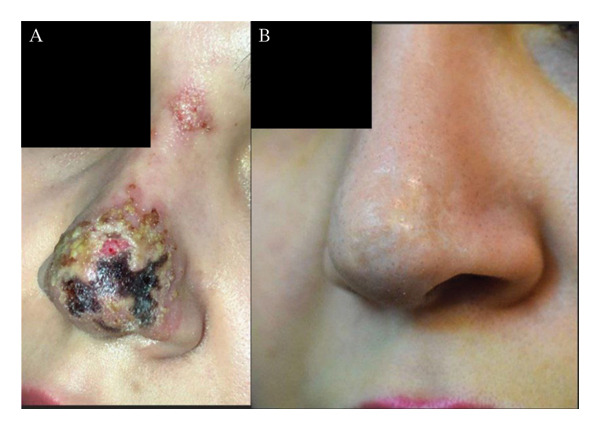
(A) Necrosis affecting the nasal tip and both soft triangles following a triamcinolone injection postrhinoplasty in a young female patient. (B) Scar formation after treatment with erbium and CO_2_ laser therapy.

A 46‐year‐old female patient, who had undergone rhinoplasty five months earlier, received a triamcinolone injection in the nasal region to reduce swelling. Following the injection, she developed necrosis affecting the nasal tip and soft triangles. Subsequent treatments included erbium and CO_2_ laser therapy, along with a secondary rhinoplasty; however, these procedures resulted in scarring. The patient consulted us for scar management (Figure 3). It is important to note that the specific interventions employed during the acute phase of ischemia remain unclear.

#### 1.2.4. Case 4

**FIGURE 4:**
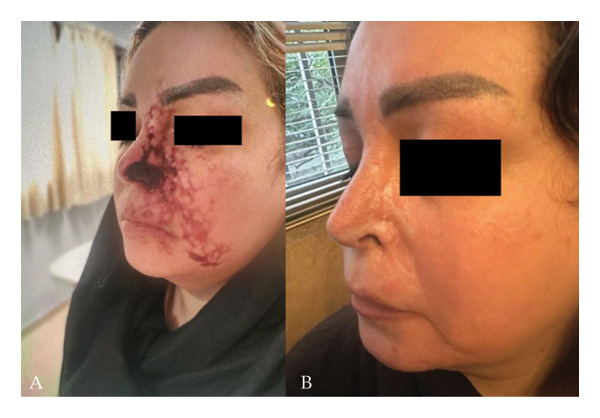
(A) Necrosis in a 41‐year‐old female following triamcinolone injection. (B) Significant scarring, and the patient seeking reconstructive surgery.

A 41‐year‐old female patient sought an online consultation for reconstructive surgery to address significant necrosis affecting the left alar rim and lateral sidewall. This complication developed after a triamcinolone injection, which led to scarring (Figure 4).

## 2. Discussion

In rare reports, the injection of triamcinolone in the head, neck, or face can obstruct blood vessels, particularly the central retinal artery, leading to central retinal artery occlusion (CRAO). Several instances of CRAO have been documented following intralesional steroid injections for conditions such as vitiligo, juvenile hemangioma [5], and keloids on the earlobe [6]. Other reported conditions associated with CRAO include injections for temporomandibular joint ankylosis [7], chronic sinusitis [8], and thyroid‐associated ophthalmopathy [9]. A notable case of cerebral infarction and CRAO occurred following a steroid injection into the forehead, highlighting the retrograde embolic potential of forceful injections [4].

Glucocorticoid particles vary in size from 1 to 1000 μm, often surpassing the average caliber of retinal arterioles and venules [10, 11]. In comparison, the inner diameter of most facial arteries typically ranges from 0.3 to 1.5 mm, making it possible for the corticosteroid particles to occlude these vessels [12].

Corticosteroid injection into the nose following rhinoplasty is a standard procedure performed one to several months postoperatively. A primary indication for this injection is the prevention or treatment of pollybeak deformity, an unpredictable complication after rhinoplasty [13]. This deformity may arise from excessive cartilage removal or insufficient resection of dorsal septal cartilage, leading to fibrosis and scarring in the subcutaneous layer of the supratip region [14]. Triamcinolone acetonide injections are commonly used in the supratip area to address this issue [15].

A 40‐day follow‐up study involving 42 patients who received 0.1 to 0.2 mL of diluted triamcinolone acetonide showed reduced skin thickness. While some studies suggest that nasal steroid injections may lead to retinal atrophy [8], others report the use of nasal steroids for both cosmetic and therapeutic purposes in sarcoidosis to be safe and effective [16]. In a study of 127 patients who received a 10‐mg/mL triamcinolone injection after nasal surgery, 85% reported improved symptoms without complications [17]. Moreover, recent reviews have reported several cases of acute vision loss following the triamcinolone injection. For instance, Park and Barmettler described 49 patients (56 eyes) with steroid‐induced vision loss, predominantly following nasal injections (45%), with involvement of the ophthalmic artery (53%) or central retinal artery (33%) [18]. In the dermatologic field, 13 cases of steroid‐induced vision loss have also been reported, emphasizing the need for meticulous attention when injecting conditions potentially associated with high‐pressure injection, such as hemangiomas, alopecia areata, scars, and keloids, particularly in high‐risk areas including the scalp, face, and periorbital region [19].

Bosma et al. reported a case of ischemia affecting three fingers following an intra‐arterial injection into the ulnar artery. The patient responded well to vasodilators, with cold intolerance being the main complication [3]. Additionally, two other cases of temporary ischemic changes in digits were documented following triamcinolone injection for CTS. These cases underscore the vasospastic reaction that may follow corticosteroid administration [20, 21]. Nicolau syndrome, presenting as acute limb ischemia following dexamethasone injection, has also been reported [22]. Furthermore, a patient experienced Nicolau syndrome symptoms following an intramatricial triamcinolone injection for nail lichen planus [23].

Prednisolone, methylprednisolone, and triamcinolone administration can lead to complete obstruction of arterioles and venules. The reduction in microvascular blood flow is primarily caused by red blood cell (RBC) aggregation, which forms spiculated RBCs [24]. Additionally, a case of eyelid necrosis following triamcinolone injection for the treatment of capillary hemangioma in a four‐week‐old girl has been reported [25]. Skin ischemia following corticosteroid administration may be an underreported condition. To our knowledge, this is the first report of triamcinolone‐induced nasal ischemia.

In the management of ischemia induced by non‐HA materials, a multimodal approach should be employed to address this emergent condition. The “THIS and FAT” protocol has been used to manage filler‐induced ischemia with satisfactory results. THIS and FAT stands for toxins (botulinum neurotoxin), high‐dose HYAL, iPRF, sPRF, antibiotics, nanofat injection, debridement, and fat membrane dressing [4]. In the present study, our cases were managed with a specialized approach that included HYAL, botulinum toxin Type A, iPRF, sPRF, and HBOT. However, our protocol has continued to evolve with subsequent cases. We now prefer to replace HBOT with nanofat as part of the “THIS and FAT” protocol, which we currently use for both filler‐related and triamcinolone‐related nasal ischemia [4]. Notably, the approach described in this series retains the practical value, particularly in settings where nanofat is unavailable or cannot be used.

Lactic acid is produced during ischemia as a result of anaerobic metabolism, leading to muscle spasms and exacerbation of the ischemic condition. Moreover, another proposed mechanism contributing to ischemia is constriction of the choke vessels, which limits collateral circulation and restricts tissue perfusion [26].

Botulinum neurotoxin (BoNT) injections are employed to induce dilation of these choke vessels and improve tissue perfusion [27]. Several studies have reported the antispasmodic effects of BoNT‐A, which enhance the necrosis process. BoNT has shown efficacy in ischemia following hand trauma by affecting soluble *N*‐ethylmaleimide‐sensitive factor attachment protein receptor (SNARE) proteins and reducing the secretion of acetylcholine, substance P, norepinephrine, calcitonin gene‐related peptide (CGRP), and glutamate [28]. BoNT promotes tissue repair and alleviates ischemia through its antivasoconstrictive properties. These characteristics stem from its ability to reduce calcium sensitivity by influencing key pathways, such as endothelial nitric oxide synthase (eNOS), cyclic guanosine monophosphate (cGMP), and soluble guanylate cyclase (sGC) [29]. Several studies have shown favorable outcomes with BoNT in patients with Raynaud’s syndrome, with clinical improvement occurring as quickly as 30 min after BoNT administration in ischemic conditions. BoNT prevents sympathetic vasoconstriction of the vascular wall muscles and blocks epinephrine neurotransmission. It also affects alpha 2C receptors, reducing the activity of chronically upregulated c‐fiber nociceptors, leading to decreased muscle spasms and pain [28, 30]. We recommend initially injecting BoNT into the ischemic areas to improve tissue perfusion by blocking vasoconstriction of the vasculatures and choke vessels.

Unlike HA fillers, which can be dissolved with HYAL, triamcinolone has no solvent. HYAL enhances tissue permeability and may improve blockages in blood vessels caused by substances other than HA [31]. Moreover, in ischemic patients, HYAL reduces edema and increases tissue permeability. It has demonstrated positive effects on electrocardiogram (ECG) changes, suggesting its potential effectiveness in treating vascular occlusion caused by triamcinolone [32, 33]. HYAL can be diluted with lidocaine or normal saline. Optimal performance has been reported at a pH range of 4.5–7.5, which falls within the normal saline and lidocaine range. Several advantages of lidocaine over normal saline include reducing pain in ischemic patients and its vasodilatory properties [34]. In several consensus statements, including the Complications in Medical Aesthetics Collaborative protocol, 1%‐2% lidocaine has been proposed as an appropriate solvent for HYAL in the treatment of filler‐induced ischemia. Accordingly, we used 1 mL of 2% lidocaine for HYAL injection [35]. In several case reports, HYAL has also been successfully used for the treatment of non‐HA‐induced necrosis [36]. Based on our expert opinion, we additionally recommend the use of HYAL in non‐HA‐related ischemic complications.

HBOT enhances tissue oxygenation, improving ischemia. HBOT activates stem cells and promotes angiogenesis, thereby assisting in the repair of damaged tissue. It also reduces swelling, combats infection, and improves recovery, protecting tissues from further damage [37–40]. Treatment with 2‐3 ATM HBOT, along with approximately 30 sessions of HBOT, and the administration of iPRF and sPRF, substantially improved ischemia [32]. Platelet concentrates continuously release high levels of growth factors and cytokines over weeks. Activation of platelets leads to the secretion of critical growth factors and proteins, including vascular endothelial growth factor (VEGF), transforming growth Factor‐β1 (TGF‐β1), bone morphogenetic proteins (BMPs), transforming growth Factor‐β2 (TGF‐β2), insulin‐like growth factor (IGF), and platelet‐derived growth factor (PDGF) [41, 42]. Altogether, PRF injection and dressing may improve the ischemic condition through the release of the aforementioned growth factors, as well as enhanced nutrient delivery and regenerative mediators that may support angiogenesis, tissue repair, and wound healing in the ischemic tissue.

The main limitation of this case series is the lack of complete demographic, procedural, and treatment‐related data for all patients, as three of the four cases were managed through remote online consultation. Another limitation is the small sample size, which limits the generalizability of the findings regarding the efficacy and safety of this multimodal protocol for triamcinolone‐induced ischemia. Further prospective studies with larger cohorts are needed to validate these preliminary observations.

Treatment of triamcinolone‐induced ischemia is similar to that of HA‐induced ischemia, although no specific solvent equivalent to HYAL exists. Based on our experience, the recommended approach for managing triamcinolone‐induced ischemia involves a combination of BoNT injections, HYAL, HBOT, iPRF, sPRF, and prophylactic antibiotics.

## 3. Conclusion

In these cases, we treated triamcinolone‐induced nasal ischemia using a combination of HYAL, BoNT A, iPRF, sPRF, and HBOT with satisfactory clinical outcomes. Since completing this case series, we have further refined our protocol by incorporating nanofat replacing HBOT as part of the “THIS and FAT” protocol, which we now apply for both filler‐ and triamcinolone‐induced nasal ischemia. Nevertheless, the original approach described here (with using HBOT without nanofat) still represents a practical and effective therapeutic strategy in conditions where nanofat is contraindicated.

## Author Contributions

S.N., N.H., and F.B. were involved in the diagnosis and management of the patient and have been responsible for the clinical part of the manuscript. M.R.P., N.H., S.O.K., M.D., and N.F‐G. did a literature review and drafted the manuscript. S.N., N.F‐G., and M.R.P. were responsible for final editing of the manuscript.

## Funding

None of the authors received any funding or financial support for the content of this article.

## Disclosure

All authors have read and approved the final manuscript.

## Conflicts of Interest

Nabil Fakih‐Gomez is a consultant for Merz Aesthetics (Frankfurt, Germany). The other authors declare no conflicts of interest.

## Data Availability

Data sharing is not applicable to this article as no datasets were generated or analyzed during the current study.
